# With Appreciation

**DOI:** 10.1002/jcsm.12812

**Published:** 2021-10-10

We thank the following individuals who served as manuscript reviewers for The Journal of Cachexia, Sarcopenia and Muscle (JCSM) in 2021.

We highly appreciate their efforts.

Fair, conscientious and timely peer reviews contribute to the success of JCSM.

The Editors.

## 6 and more reviews

Matthew Alexander

Stephen Alway

Anjalee Thanuja Amarasekera

Richard Bohannon

Andrea Bonetto

T. Scott Bowen

Jeffrey Brault

Andrew Coats

Paola Costelli

Gangarao Davuluri

Rosario Donato

Stephanie Duguez

Vandre Figueiredo

Giacomo Garibotto

Marcus Goncalves

Craig Goodman

Nicholas Greene

Zhaoyong Hu

Joshua Huot

Satoshi Ikeda

Masaaki Konishi

Siegfried Labeit

Barry Laird

Alessandro Laviano

Markus Luedi

Frank Misselwitz

Zudin Puthucheary

Kunihiro Sakuma

Mattia Scalabrin

Joerg Schefold

Ashley Smuder

Hidetaka Wakabayashi

## 4–5 reviews

Naoki Akazawa

Markus Anker

W. David Arnold

Vickie E Baracos

Juergen Bauer

Bert Blaauw

Robert Bloch

Daniel Cannon

Joe Chakkalakal

Andrew Clark

Maria Conte

Lance Davidson

Marcelo Dos Santos

Guilherme Fonseca

Russell Hepple

Alp Ikizler

Aminah Jatoi

Serkan Kir

Oliver Klein

Alexander Krannich

Stephanie Krasnow

Maciej Lalowski

Paul Lewandowski

Xiaolin Li

Johanna Magga

Tateaki Naito

Hiroki Nishikawa

Ismail Ozsoy

Marcelo Pereira

Rosanna Piccirillo

Wagner Ripka

Masakazu Saitoh

Patrick Schober

Ng Shyh‐Chang

Yoshihiro Yoshimura

## 2–3 reviews

Yasser Abdelhafez

Hidenori Arai

Anne‐Sophie Armand

Saro Armenian

Atsushi Asakura

Azeddine Atfi

Asfar Azmi

Charlotte Beaudart

Gwenaelle Begue

Kirsten Bell

Libera Berghella

Pengpeng Bi

Laure B. Bindels

Jose‐Ramon Blanco

Sue Bodine

Federico Bozzetti

Scott Brakenridge

Marie‐Kathrin Breyer

Alain Bruhat

Olivier Bruyere

Claudio Cabello‐Verrugio

Eduardo Cadore

Federica Calore

Donny Camera

Giuseppina Caretti

James Carson

Robson Carvalho

Paolo Caserotti

Peggy Cawthon

Wah Chan

Jie Chen

Tai‐Heng Chen

Yi‐Wen Chen

Liang‐Kung Chen

Stephanie Chevalier

John Chiang

Chih‐Kang Chiang

Che‐Sheng Chu

Sina Coldewey

Dario Coletti

Giancarlo Condello

Nancy Cook

Marie Csete

Constantinos Davos

Boel De Paepe

Hans Degens

Patrice Delafontaine

Louise Deldicque

Brian Derstine

Rony Dev

Bijan Dey

Francesco Dioguardi

Jean‐Francois Dumas

Esther Dupont‐Versteegden

Mohammed Eslam

Alessandro Fanzani

Jens Fielitz

Nicoletta Filigheddu

James Fluckey

Armin Frille

Christopher Fry

Davide Gabellini

Imed Gallouzi

Jose Garcia

Tania Garfias‐Veitl

Corinna Geisler

Duska Glavas

Enrique Gomez Barrena

Mari Carmen Gomez‐Cabrera

Maria Cristina Gonzalez

Loncar Goran

Frank Graef

Zachary Graham

Mark Griffiths

Christelle Guillet

Cheng Guo

Denis Guttridge

David Hammers

Mohamed Hankir

Justin Hardee

Michiyoshi Hatanaka

Wei He

Christopher R. Heier

Nathan Hodson

Benjamin Horne

Chikwendu Ibebunjo

Tatsuro Inoue

Connie Jackaman

Rishi Jain

Li Li Ji

Krystian Josiak

Andrew R. Judge

Lin Kang

David Karasik

Carrie Karvonen‐Gutierrez

Frederick Kaskel

Takumi Kawaguchi

Seyyed Mohammad Kazemi‐Bajestani

Andy Khamoui

Dong Joon Kim

Ben Kirk

Nicole Kiss

Christian Kleber

Yoji Kokura

Hirotaka Komaba

Avinash Kumar

Ashok Kumar

Mitja Lainscak

Simon Lebech Cichosz

Lorenz Lehmann

Helmar Lehmann

Alexander Leichtle

Yi‐Ping Li

Chi‐Chien Lin

Hung‐Hsin Lin

Malene Lindholm

Hanns Lochmüller

Michael Lustgarten

Antonio Macciò

Leonardo Mancabelli

Giovanni Mantovani

Daniela Mari

Daniel Marks

Lisa Martin

Anne May

Ryan McGrath

Donald McMillan

Gretchen Meyer

Yi Miao

Cameron Mitchell

Ryo Momosaki

Paul Morgan

Keiko Motokawa

Merrilee Needham

Sagar U. Nigwekar

Hiroshi Nishimune

Enzo Nisoli

Yoshitsugu Obi

Sumito Ogawa

Koichi Okita

Jongha Park

Hyuntae Park

Jeroen Pasterkamp

Fabio Penna

Stany Perkisas

Gustavo Pimentel

Fabrizio Pin

Emidio Pistilli

Paolo Porporato

S Russ Price

Sarah Psutka

Melissa Puppa

Jens Reimann

Carlo Ricciardi

Donato Rivas

Eric Roeland

Maria Rohm

Jose Rosa Neto

Bob Roshanravan

Kotomi Sakai

Marília Seelaender

Hiroshi Shamoto

Adam Sharples

Søren Sheikh

Kengo Shirado

Shashi Singh

Richard Skipworth

Gordon Smith

Athanassia Sotiropoulos

Espen Spangenburg

Pietro Spitali

Elani Streja

Cynthia Stretch

Veedamali Subramanian

Arnold Suda

Ken Sugimoto

Mizue Suzuki

Peter Szentesi

Murad Taani

Yasuharu Tabara

Marine Theret

Jean‐Paul Thissen

David Thomas

Malte Tiburcy

Andrea Ticinesi

Michael Toth

Trupti Trivedi

Bruce Troen

Shigeru Tsunoda

Junko Ueshima

Holly Van Remmen

Sjors Verlaan

Stefano Volpato

Christine von Arnim

Peiyu Wang

Amal Wanigatunga

Steven Welc

Yefei Wen

Saffron Willis‐Owen

Carol Witczak

Chang Won Won

Jean Woo

Rob Wust

Junjie Xiao

Hong Xia Xu

Qian‐Li Xue

Yosuke Yamada

Chunzhang Yang

Mee‐Sup Yoon

Werner Z'Graggen

Ruoyu Zhang

Yao Zhu

Cheng‐le Zhuang

Jeffrey Zigman

Teresa Zimmers

Carmine Zoccali

